# Proteomic Profiling of Autophagosome Cargo in *Saccharomyces cerevisiae*

**DOI:** 10.1371/journal.pone.0091651

**Published:** 2014-03-13

**Authors:** Kuninori Suzuki, Shingo Nakamura, Mayumi Morimoto, Kiyonaga Fujii, Nobuo N. Noda, Fuyuhiko Inagaki, Yoshinori Ohsumi

**Affiliations:** 1 Bioimaging Center, Graduate School of Frontier Sciences, University of Tokyo, Kashiwa, Chiba, Japan; 2 Department of Structural Biology, Faculty of Advanced Life Science, Hokkaido University, Sapporo, Hokkaido, Japan; 3 Laboratory of Molecular Structure, Institute of Microbial Chemistry (BIKAKEN), Tokyo, Japan; 4 Frontier Research Center, Tokyo Institute of Technology, Yokohama, Kanagawa, Japan; Cambridge University, United Kingdom

## Abstract

Macroautophagy (autophagy) is a bulk protein-degradation system ubiquitously conserved in eukaryotic cells. During autophagy, cytoplasmic components are enclosed in a membrane compartment, called an autophagosome. The autophagosome fuses with the vacuole/lysosome and is degraded together with its cargo. Because autophagy is important for the maintenance of cellular homeostasis by degrading unwanted proteins and organelles, identification of autophagosome cargo proteins (i.e., the targets of autophagy) will aid in understanding the physiological roles of autophagy. In this study, we developed a method for monitoring intact autophagosomes *ex vivo* by detecting the fluorescence of GFP-fused aminopeptidase I, the best-characterized selective cargo of autophagosomes in *Saccharomyces cerevisiae*. This method facilitated optimization of a biochemical procedure to fractionate autophagosomes. A combination of LC-MS/MS with subsequent statistical analyses revealed a list of autophagosome cargo proteins; some of these are selectively enclosed in autophagosomes and delivered to the vacuole in an Atg11-independent manner. The methods we describe will be useful for analyzing the mechanisms and physiological significance of Atg11-independent selective autophagy.

## Introduction

Macroautophagy, hereafter referred to as autophagy, is a system for bulk degradation of cytoplasmic materials. It is mediated by a double-membrane-bound organelle, the autophagosome. Bulk autophagy serves to maintain the intracellular pool of free amino acids that is necessary for protein synthesis under nutrient-starvation conditions [1].

Autophagy is predominantly a non-selective degradation system, but several selective cargoes have recently been reported. Two modes of selective autophagy have been proposed, one dependent on Atg11 (autophagy-related 11) and the other independent of this protein [2]. The former mode is relatively more selective; some vacuolar proteins are constitutively transported to the vacuole by Atg11-dependent selective autophagy under nutrient-rich conditions. This pathway, termed the cytoplasm-to-vacuole targeting (Cvt) pathway, transports the Cvt complex to the vacuole [3]. The major component of the Cvt complex is vacuolar aminopeptidase I (Ape1), which is synthesized as a proform (prApe1) and forms the core structure of the Cvt complex. After transport to the vacuole, prApe1 proteolytically matures (mApe1). By contrast, abundant cytoplasmic components, such as cytoplasmic aldehyde dehydrogenase (Ald6) and ribosomes, are degraded by Atg11-independent selective autophagy. In particular, degradation of Ald6 helps to maintain the redox state of the cytoplasm and is therefore physiologically important for cell survival under starvation [4]. Selective degradation of ribosomes, termed ribophagy, involves a ubiquitin-specific protease Ubp3 and its regulatory subunit Bre5 [5]. Moreover, binding partners of the Ubp3/Bre5 complex, a chaperone-like protein Cdc48 and a ubiquitin-binding protein Ufd3, are required for ribophagy [6]. Because ribophagy is independent of Atg19 [5], it is unlikely that Atg11 is important for this pathway. To understand the physiological significance of autophagy, comprehensive identification of autophagosome cargo is required.

Disruption of *YPT7*, which encodes a GTPase required for homotypic fusion of vacuoles, causes autophagosomes to accumulate in the cytoplasm, because the fusion of autophagosomes with the vacuole is prevented [7]. The presence of autophagosomes in *ypt7*Δ cells is determined biochemically using a proteinase K–protection assay [8], in which immunoblot analysis is used to detect proteinase K–resistant prApe1 inside autophagosomes. Cell fractionation analysis revealed that autophagosomes are collected in the 15,000×*g* pellet fraction [8]. However, autophagosomes in the yeast *Saccharomyces cerevisiae* have never been identified *ex vivo* by microscopy.

In this study, we first developed a simple and easy method for detecting intact autophagosomes *ex vivo*, which facilitated optimization of a biochemical procedure for fractionating autophagosomes. LC-MS/MS and subsequent statistical analyses of autophagosome fractions yielded a catalog of autophagosome cargoes.

## Materials and Methods

### Yeast Strains and Growth Conditions

The yeast strains used in this study are listed in Table 1. Standard protocols were used for yeast manipulation [9]. The strain overexpressing N-terminally GFP-fused prApe1 was generated using a PCR-based gene-modification method [10]. Cells were grown in YEPD medium (1% yeast extract, 2% peptone, 2% glucose). Autophagy was induced by transferring cells to nitrogen-starvation SD(-N) medium (0.17% yeast nitrogen base w/o amino acids and ammonium sulfate, 2% glucose).

**Table 1:** Strains used in this study.

Strain	Genotype	Source
SEY6210	*MATα lys2 suc2 his3 leu2 trp1 ura3*	[26]
KVY4	SEY6210; *ypt7*Δ*::LEU2*	[7]
YAK1	SEY6210; *ypt7*Δ*::HIS3 atg1*Δ*::LEU2*	Lab stock
KVY55	SEY6210; *pho8*Δ*::P_TDH3_-pho8*Δ*60*	[8]
GYS775	KVY55; *ape1*Δ*::natNT2-P_GPD1_-GFP-APE1*	This study
GYS776	GYS775; *ypt7*Δ*::HIS3*	This study
GYS781	GYS775; *ypt7*Δ*::HIS3 atg1*Δ*::LEU2*	This study
YCK414	GYS775; *ypt7*Δ*::HIS3 atg11*Δ*::LEU2*	This study
TVY1	SEY6210; *pep4*Δ*::LEU2*	[3]
GYS376	SEY6210; *pep4*Δ*::LEU2 atg7*Δ*::HIS3*	This study

### Fluorescence Microscopy

Fluorescence microscopy was performed using an IX71 TIR-FM system (Olympus) equipped with a UPlanSApo100×Oil (NA: 1.40) and a CoolSNAP HQ CCD camera (Nippon Roper, Japan), as described [11]. For observation of GFP, a blue laser (Sapphire 488-20, Coherent) was used for excitation. For simultaneous observation of GFP and R18 (octadecyl rhodamine B, Invitrogen), fluorescence emission excited by blue and yellow (85-YCA-010, Melles Griot) lasers were split with a U-SIP (Olympus). Images were acquired and processed using the MetaMorph software (Molecular Devices).

For R18 staining, samples were stained with 10 µg/ml of R18 (1 mg/ml stock dissolved in dimethyl sulfoxide) for 10 minutes at room temperature. The samples were observed after they were washed twice with fresh buffer.

### Electron Microscopy

Electron microscopy was performed by Tokai-EMA, Inc. (Japan) as described [12]. Briefly, samples were harvested by centrifugation, and the resulting pellets were sandwiched between copper grids and rapidly frozen in liquid propane using a Leica EM CPC cryofixation system (Leica). For ultrastructural analysis, samples were freeze-substituted in 2% osmium tetroxide dissolved in acetone and embedded in Quetol 651 resin. For immunoelectron microscopy, samples were freeze-substituted in acetone containing 1–3% distilled water and embedded in LR White resin. Anti-Ape1 antiserum [13] was used to detect Ape1.

### Proteinase K–protection Assay

Cells grown to OD_600_ = ∼1.5 in 50 ml of YEPD medium were starved in 50 ml of SD(-N) medium. Collected cells were converted to spheroplasts with Zymolyase 100T, and then mechanically disrupted with 3.0 µm–pore polycarbonate filters (Whatman) in lysis buffer (HES_1.0_ buffer; 20 mM HEPES [pH 7.5], 5 mM EDTA, 1 M sorbitol). After cell debris was removed, lysates were divided into aliquots and treated with or without 1% Triton X-100 (Nacalai Tesque, Japan), 1 mg/ml proteinase K (Roche), or both for 30 minutes on ice. The reactions were terminated by addition of 20% trichloroacetic acid (TCA). Precipitates were washed with acetone and dissolved in SDS-PAGE sample buffer containing 4 mM Pefabloc SC (Roche). Equivalent quantities of protein were subjected to immunoblot analysis.

### Continuous Density Gradient Analysis

Cell lysates were prepared as described above and centrifuged at 15,000×*g* for 15 minutes at 4°C. The pellets were resuspended in 500 µl HES_1.0_ buffer, and then layered onto 10 ml of 0–30% continuous iodixanol gradients using Optiprep (Axis-Shield), which is a solution of 60% iodixanol in water. The gradients were constructed in 13 PA tubes (HITACHI, Japan) using a Gradient Master gradient maker (Biocomp Instruments). Loaded gradients were ultracentrifuged at 100,000×*g* for 20 hour at 4°C in a P40ST rotor on a CP-80β ultracentrifuge (HITACHI, Japan). Twenty-four fractions (approximately 440 µl each) were taken from each tube, starting from the top of the gradient. Each fraction was mixed with 70 µl of 100% TCA, and then incubated for 15 minutes on ice. The TCA-precipitated fractions were centrifuged at 15,000×*g* for 10 minutes at 4°C in a T15AP31 rotor on a CF15RX centrifuge (HITACHI, Japan). The pellets were washed once with 150 µl of ice-cold acetone using a bath sonicator (D-SONIC; SND, Japan). After the pellets were centrifuged at 15,000×*g* for 5 minutes at 4°C, the acetone was discarded and the samples were dried using a VC-15SP centrifugal concentration apparatus (TAITEC, Japan). The pellets were dissolved in 70 µl of SDS-PAGE sample buffer, and 10-µl aliquots were subjected to immunoblot analysis.

### Immunoblot Analysis

Primary antibodies used were anti-Ape1, anti-Pgk1 (A6457, Invitrogen), anti-Rpl17 (generous gift from Dr. Sabine Rospert, University of Freiburg, Germany) [14], anti-Atg8 [7], anti-Mge1 (generous gift from Dr. Andreas Reichert, Goethe University Frankfurt am Main, Germany), anti-Dpm1 (A6429, Invitrogen), anti-Van1 (generous gift from Dr. Koji Yoda, University of Tokyo, Japan), anti-Pep12 (A21273, Invitrogen), anti-Pfk (generous gift from Dr. Jürgen J. Heinisch, University of Leipzig, Germany) [15], anti-Adh [16], and anti-Ald6 [4]. Horseradish peroxidase–conjugated antibodies were used as secondary antibodies. Chemiluminescence signals produced by an ECL reagent (Western Lightning Plus-ECL, PerkinElmer; ECL Prime Western Blotting Detection System, GE healthcare) were detected using a CCD camera system (LAS, Fujifilm, Japan).

### Procedure to Obtain Autophagosome Fractions for Mass Spectrometry

Cells were grown to OD_600_ = ∼1.5 in 1 L YEPD medium at 30°C, washed once with distilled water, and starved in 300 ml of SD(-N) medium for 3 hours. Collected cells were suspended in 40 ml of pre-spheroplasting buffer (100 mM Tris-HCl [pH 9.0], 40 mM β-mercaptoethanol) and incubated for 10 minutes at 30°C. The cells were collected by centrifugation at 2,000×*g* for 2 minutes in a TS-7LB rotor on a LX-120 centrifuge (TOMY SEIKO, Japan). The pelleted cells were suspended in 8 ml of spheroplasting buffer (20 mM Tris-HCl [pH 7.5], 1.4 M sorbitol) containing 1 mg/ml Zymolyase 100T (Seikagaku-kogyo, Japan), and the resultant suspension was diluted with 32 ml of spheroplasting buffer (final volume, 40 ml). The cells were converted to spheroplasts by incubation for 25 minutes at 30°C with gentle shaking. Spheroplasts were collected by centrifugation at 1,000×*g* at 4°C, washed twice with 40 ml of 1.4 M sorbitol, resuspended in 40 ml of HES_1.0_ buffer, and then mechanically disrupted with 3.0 µm–pore polycarbonate filters. After cell debris was removed by centrifugation at 300×*g* for 1 minute at 4°C, cell lysates were passed through 2.0 µm–pore polycarbonate filters (Whatman). The lysates were again centrifuged at 500×*g* for 1 minutes at 4°C and the cleared lysates were centrifuged at 15,000×*g* for 15 minutes at 4°C. The pellets were suspended in 900 µl of HES_1.0_ buffer, and then 100 µl of 10 mg/ml proteinase K dissolved in HES_1.0_ buffer was added. This mixture was incubated at 37°C for 30 minutes; reactions were terminated by addition of 10 µl of 400 mM Pefabloc SC dissolved in HES_1.0_ buffer, and then filtered through 0.8 µm–pore polycarbonate filters (Whatman). Samples were layered onto discontinuous iodixanol gradients (1.5 ml of 20%; 6 ml of 10%; 4 ml of 5%), which were constructed in 13.2-ml polyallomer centrifuge tubes (331372, Beckman Coulter). Loaded gradients were ultracentrifuged at 100,000×*g* for 60 minutes at 4°C in a SW41Ti rotor on an Optima L-80 XP ultracentrifuge (Beckman Coulter). GFP fluorescence was visualized using a blue LED light with a band-pass filter (Optocode, Japan). Bands at the 10–20% interface were taken with Pasteur pipettes (500–1000 µl each) and precipitated with 1/40 volume of 20% Triton X-100 (final 0.5%) and 1/5 volume of 100% TCA (final 20%). The pellets were washed twice with 600 µl of ice-cold acetone and dissolved in 50 µl of phosphate-buffered saline containing 8 M urea (2–5 mg protein/ml). Protein concentrations were measured using the BCA Protein Assay reagent (Thermo Scientific).

### LC-MS/MS Spectrometry

Methods for LC-MS/MS spectrometry are described in Appendix S1.

### Principal Component Analyses (PCA)

We started with a matrix of mass spectrometric data, with each row corresponding to a different protein and each column corresponding to the number of spectral counts detected by LC-MS/MS in each fraction (Table S1). First, 378 proteins with more than one spectral count in all *ypt7*Δ autophagosome fractions were selected from 673 proteins (Table S2). Second, these parameters were subjected to PCA using data sets for autophagosome fractions (*ypt7*Δ, *ypt7*Δ*atg1*Δ, and *ypt7*Δ*atg11*Δ cells) and for cytosol (100,000×*g* supernatant) prepared from *ypt7*Δ cells (gray-labeled parameters in Table S3) to extract the principal components (PCs). The first two PCs account for about 95% of the total variability (Table S3). Third, localization of each protein was manually annotated as three localizations (mitochondrial, plasma membrane, or other cytoplasmic proteins) based on the *Saccharomyces* Genome Database (http://www.yeastgenome.org/) (Table S4). Finally, the PC scores were plotted.

To analyze the cytoplasmic proteins further, 186 proteins with PC2 scores greater than or equal to zero were selected and subjected to PCA using data sets for autophagosome fractions (Table S5). Because Tdh3 and prApe1 had large impacts on the results of PCA, the data for these proteins were omitted from further analysis. Next, the first and second PCs were calculated, using data sets for *ypt7*Δ and *ypt7*Δ*atg1*Δ autophagosome fractions (Table S6), and then plotted. All statistical analyses were performed using Microsoft Excel 2007 with the Solver add-in (Microsoft).

## Results

### Visualization of Autophagosomes in Cell Lysates by Microscopy

**Figure 1:**
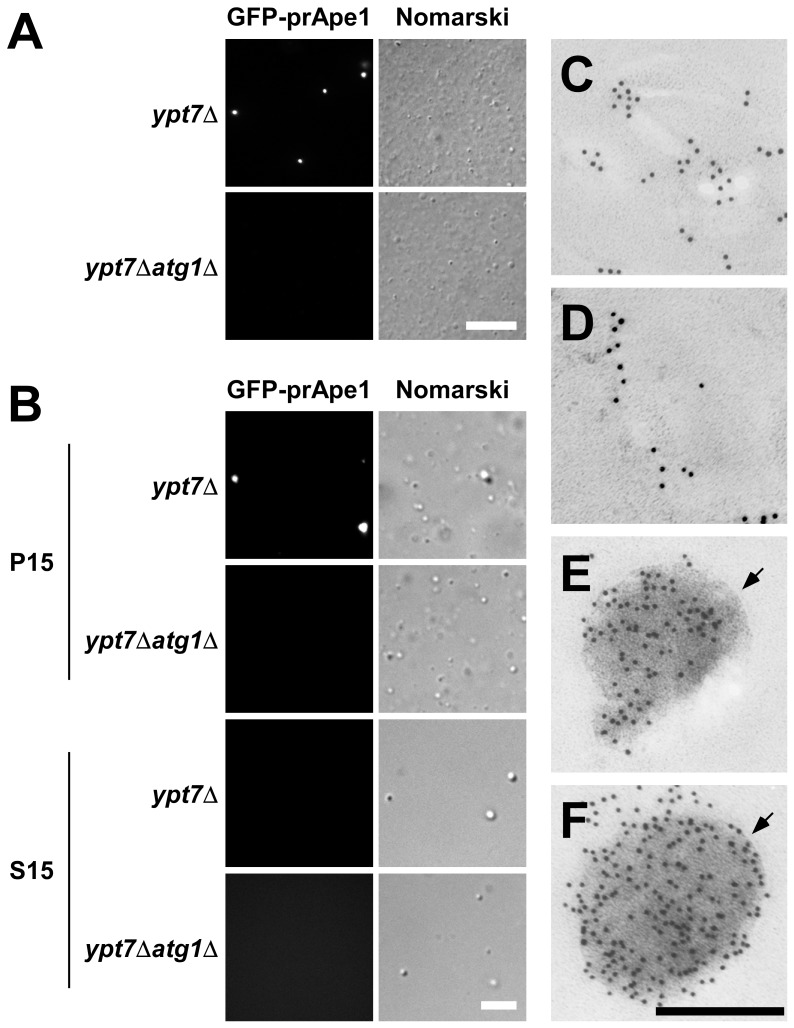
Visualization of autophagosomes in cell lysates by microscopy. (A) Total cell lysates from cells grown in YEPD medium and starved in SD(-N) medium for 4.5 hours. Spheroplasts were lysed with a 3.0 µm–pore filter, and cell debris was removed. Each fluorescence image of GFP-prApe1 was taken with the same exposure time. Bar represents 10 µm. (B) Total cell lysates were centrifuged at 15,000×*g* and separated into pellet (P15) and supernatant (S15) fractions. Each fluorescence image of GFP-prApe1 was taken with the same exposure time. Bar represents 5 µm. (C–F) Ultrathin sections were probed with an anti-Ape1 antiserum and observed by electron microscopy. (C) P15 fraction from the *ypt7*Δ*atg1*Δ strain. (D) Background labeling in the P15 fraction of *ypt7*Δ cells. (E–F) Autophagosomes in the P15 fraction from *ypt7*Δ cells. Arrows indicate autophagosome membranes. Bar indicates 200 nm.

We prepared the 15,000×*g* pellet (P15) fraction from nitrogen-starved *ypt7*Δ cells and subjected the fraction to immunoelectron microscopy using an anti-Ape1 antiserum. Although numerous membrane structures were non-specifically labeled, we could not identify autophagosomes (data not shown). We overexpressed GFP-prApe1 in *ypt7*Δ cells to increase the signal in fluorescence and immunoelectron microscopy. We then monitored fluorescence of GFP-prApe1 by fluorescence microscopy. Under nitrogen-starvation conditions, only one bright dot was seen in *ypt7*Δ*atg1*Δ cells (data not shown), suggesting that this dot corresponded to the Ape1 complex localized outside autophagosomes [13], [17], whereas in *ypt7*Δ cells, several weaker dots were seen in addition to the bright dot (Fig. S1). In cell lysates prepared from these strains, numerous vesicular structures were recognized by bright-field microscopy (Fig. 1A). Fluorescence microscopy revealed GFP-prApe1 dots in the *ypt7*Δ lysate, whereas such dots could hardly be seen in the *ypt7*Δ*atg1*Δ lysate (Fig. 1A). This result suggests that the dots seen in the *ypt7*Δ lysate correspond to autophagosomes, and that GFP-prApe1 dots outside autophagosomes disperse upon exposure to the lysis buffer.

These GFP-Ape1 dots were collected in the P15 fraction but not in the 15,000×*g* supernatant (S15) fraction (Fig. 1B). We observed the P15 fractions obtained from *ypt7*Δ and *ypt7*Δ*atg1*Δ cells by immunoelectron microscopy, using an anti-Ape1 antiserum. In the P15 fraction of *ypt7*Δ*atg1*Δ cells, only membranous structures were labeled (Fig. 1C). By contrast, in the P15 fraction of *ypt7*Δ cells, electron-dense and spherical structures labeled by the antiserum, in addition to the background labeling (Fig. 1D), were detected (Fig. 1E–F). Furthermore, autophagosome membranes were detected around the spherical structures (arrows in Fig. 1E–F). As far as we know, this is the first report of morphological detection of autophagosomes *ex vivo* by immunoelectron microscopy using an autophagosome-specific marker.

**Figure 2:**
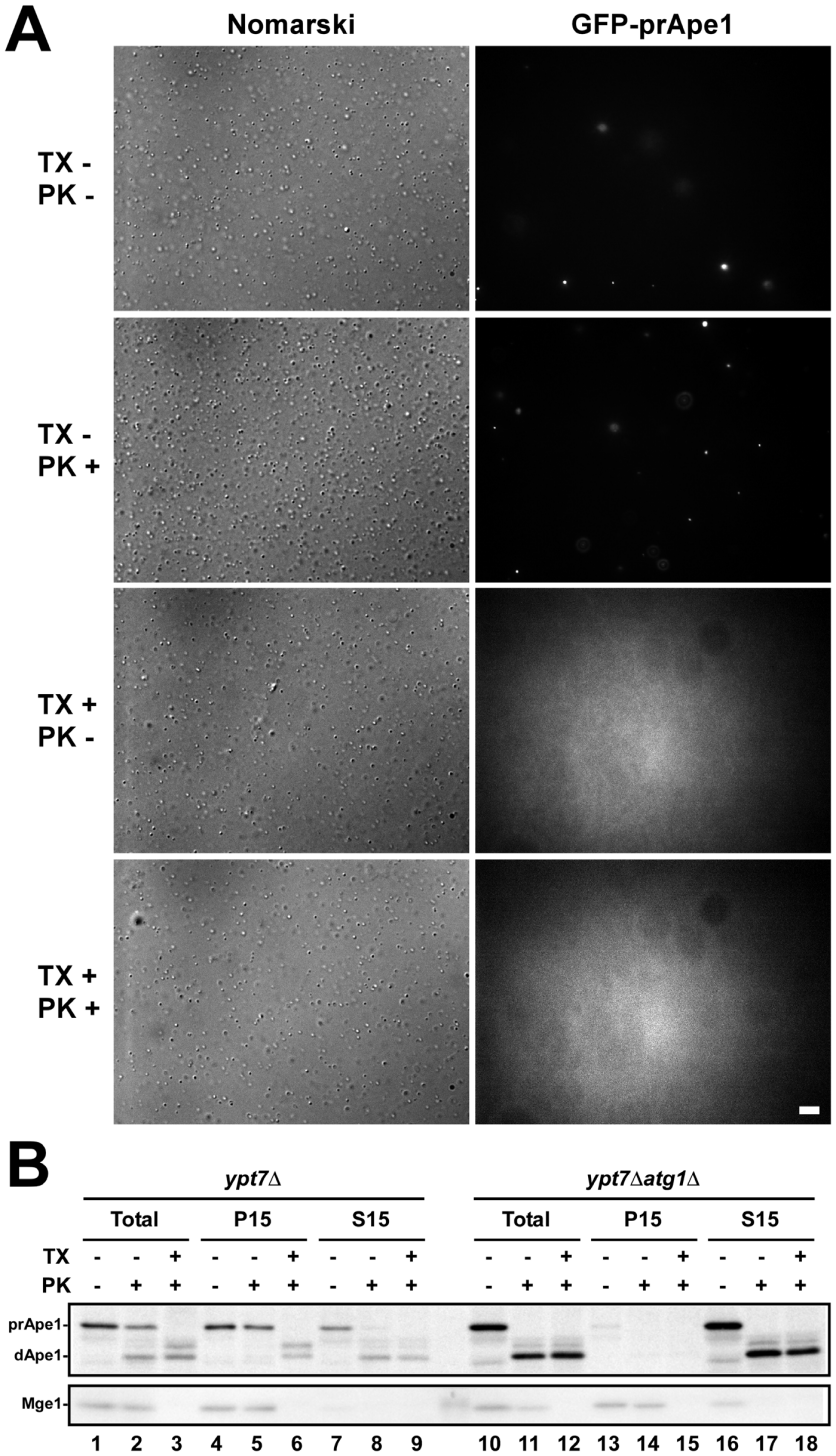
GFP-prApe1–labeled dots correspond to intact autophagosomes. (A) The 15,000×*g* pellet (P15) fraction from *ypt7*Δ cells was treated with 1% Triton X-100 (TX), 0.5 mg/ml proteinase K (PK), or both for 30 minutes at 37°C before observation by fluorescence microscopy. Each fluorescence image of GFP-prApe1 was taken with the same exposure time. Bar represents 5 µm. (B) Detection of autophagosome by proteinase K-protection assay. Autophagosomes were collected into the P15 fraction (lanes 4–6). In *ypt7*Δ*atg1*Δ cells, all prApe1 was collected into the 15,000×*g* supernatant (S15) fraction (lanes 16–18). TX represents the presence of 1% Triton X-100, PK indicates the presence of 1 mg/ml proteinase K. prApe1 and dApe1 indicate precursor Ape1 and degraded Ape1, respectively. Mge1 is a mitochondrial matrix protein.

Next, we treated the P15 fraction from *ypt7*Δ cells containing GFP-prApe1 dots with proteinase K, Triton X-100, or both. In the presence of Triton X-100, the GFP-Ape1 dots disappeared, and the background fluorescence increased (Fig. 2A), indicating that GFP-prApe1 dots exposed to the buffer diffuse when autophagosome membranes are solubilized by detergent. We confirmed that prApe1 in the P15 fraction from *ypt7*Δ cells was not accessible to proteinase K (Fig. 2B). We concluded that the GFP-prApe1 dots correspond to intact autophagosomes.

### Biochemical Procedure for Obtaining an Autophagosome-enriched Fraction

**Figure 3:**
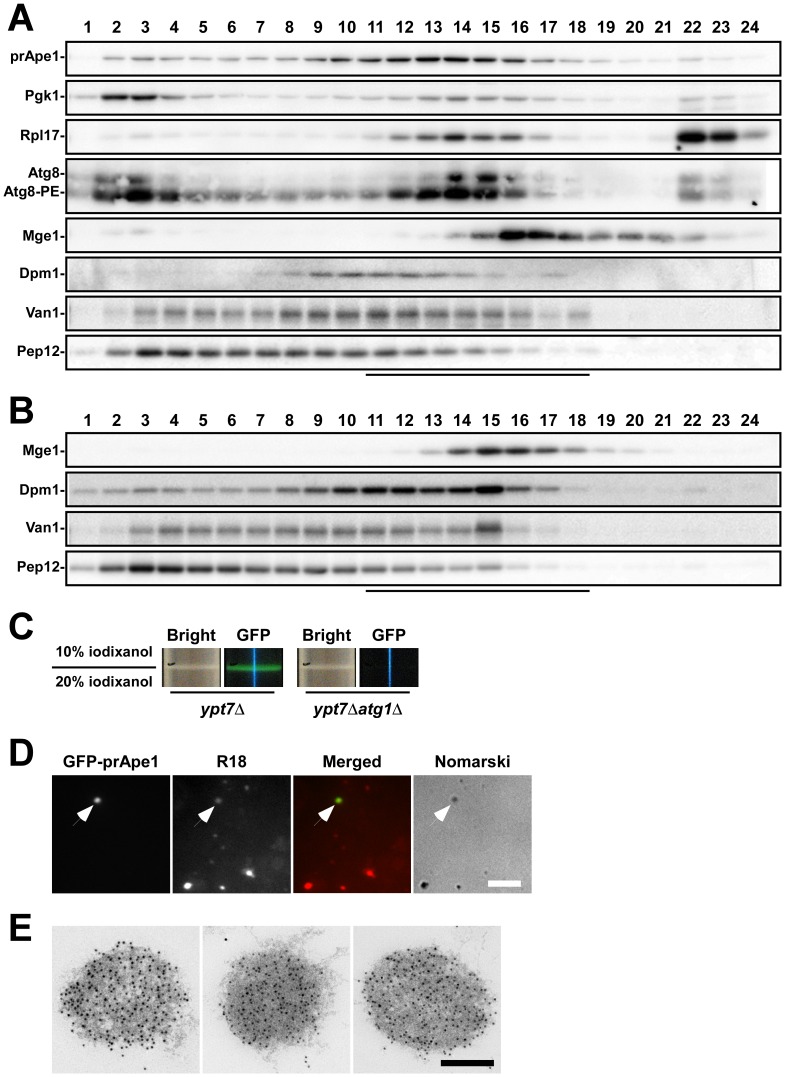
Density gradient analysis of the P15 fraction. P15 fractions from *ypt7*Δ cells (A) and *ypt7*Δ*atg1*Δ cells (B) were subjected to iodixanol density gradient centrifugation. Autophagosome markers (prApe1 and Atg8) and cytoplasmic components enclosed in autophagosomes (Pgk1, 3-phosphoglycerate kinase; Rpl17, ribosomal 60S subunit protein) were detected in fractions #11–18, which correspond to 11.6–17.8% iodixanol concentrations. Dpm1 (ER), Van1 (Golgi), and Pep12 (endosome) were used as organelle markers. Mitochondria labeled with mitochondrial-matrix protein Mge1 were detected in the same fractions in *ypt7*Δ and *ypt7*Δ*atg1*Δ cells (lines below each panel). Atg8-PE indicates phosphatidylethanolamine conjugated Atg8 [25]. (C) Magnified images of centrifuge tubes at the interface between the 10% and 20% concentrations of iodixanol after centrifugation. Bright-field and fluorescence images excited with blue light are shown. Blue vertical lines in the GFP images are reflections of blue light used for excitation. (D) Autophagosomes were detected under a fluorescence microscope. The turbid band from *ypt7*Δ cells (C) was stained with R18 and subjected to fluorescence microscopy. Autophagosomes are recognized as a weakly R18-stained dot (arrows). Bar represents 5 µm. (E) Autophagosomes detected by immunoelectron microscopy in the autophagosome fraction from *ypt7*Δ cells (C). Localization of prApe1 was detected with an anti-Ape1 antiserum. Bar represents 200 nm.

Because autophagosomes are collected in the P15 fraction of *ypt7*Δ cells, we used this fraction for further analysis. First, we treated P15 fraction with proteinase K to degrade contaminated proteins outside autophagosomes. Then, the proteinase K–treated P15 fraction was passed through 0.8 µm–pore polycarbonate filters and subsequently subjected to continuous iodixanol density-gradient centrifugation. prApe1 was collected in fractions containing 11.6–17.8% of iodixanol (Fig. 3A, lanes 11–18). In these fractions, a marker of autophagic membranes (Atg8) was also detected, indicating that these fractions contain autophagosomes. Moreover, cytoplasmic components, such as Pgk1 (3-phosphoglycerate kinase) and Rpl17 (ribosome), were also present in these fractions (Fig. 3A). These results indicate that we successfully fractionate autophagosomes containing cytoplasm. Next, we examined a fractionation pattern of mitochondria (Mge1), ER (Dpm1), Golgi (Van1), and endosomes (Pep12). These marker proteins were present in these fractions even in the absence of autophagosomes (Fig. 3B).

To collect sufficient numbers of autophagosomes for mass spectrometry, P15 fractions were separated by iodixanol discontinuous gradients. A single turbid band appeared at the interface between 10% and 20% iodixanol in samples from both *ypt7*Δ and *ypt7*Δ*atg1*∶ lysates (Fig. 3C, bright). GFP fluorescence was detected only from the *ypt7*Δ band (Fig. 3C, GFP), indicating that intact autophagosomes are included in this band. The absence of GFP fluorescence in the *ypt7*Δ*atg1*Δ band suggests that organelles other than autophagosomes are present in that band.

The band from *ypt7*Δ was stained with a lipophilic R18 dye, and then subjected to fluorescence microscopy. Dots recognized by bright-field microscopy were labeled with R18 (Fig. 3D), implying that membrane structures are enriched in these bands. GFP-prApe1 dots were detected and weakly stained with R18 (Fig. 3D), indicating that intact autophagosomes are recovered. Immunoelectron microscopy confirmed that autophagosomes were actually present in this fraction (Fig. 3E). Hereafter, we call this fraction the ‘AP (autophagosome) fraction’.

### Mass Spectrometric Analysis of the AP Fraction

We analyzed the AP fraction from *ypt7*Δ cells by LC-MS/MS. To quantify the relative abundance of each protein, we used the spectral-counting method, which is based on the fact that the number of spectral counts detected by LC-MS/MS reflects the amount of each protein [18]. prApe1 and Ald6 were detected in the top 40 proteins identified in the AP fraction of *ypt7*Δ cells (Table S7), suggesting that cargo proteins of autophagosomes can be detected by mass spectrometry. Most of the top 40 proteins detected in the AP fraction of *ypt7*Δ*atg1*Δ cells were mitochondrial proteins (Table S8), suggesting that mitochondria are enriched in this fraction; these data are consistent with the results of density-gradient analysis (Fig. 3A–B). Moreover, these observations also suggest that the majority of the mitochondrial proteins detected in the AP fraction of *ypt7*Δ cells are derived from contaminating mitochondria.

### Statistical Analysis of the Data Obtained from Mass Spectrometry

**Figure 4:**
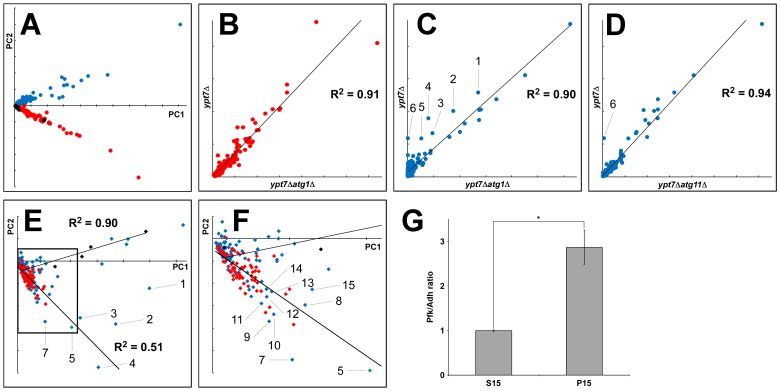
Profiling of autophagosome cargo by mass spectrometry. (A) Scatter plot of the first and second principal components (PCs) from principal component analysis (PCA). PC1 and PC2 indicate the first and second PCs, respectively. Blue, red, and black dots indicate cytoplasmic, mitochondrial, and plasma-membrane proteins, respectively. (B) Scatter diagram of mitochondrial proteins detected in AP fractions from *ypt7*Δ*atg1*Δ and *ypt7*Δ cells. (C) Scatter diagram of cytoplasmic proteins detected in AP fractions from *ypt7*Δ*atg1*Δ and *ypt7*Δ cells. (D) Scatter diagram of cytoplasmic proteins detected in AP fractions from *ypt7*Δ*atg11*Δ and *ypt7*Δ cells. (E) Scatter plot of the first and second PCs of cytoplasmic proteins in AP fractions from *ypt7*Δ and *ypt7*Δ*atg1*Δ cells. Blue, red, and black diamonds indicate cytoplasmic, ribosomal, and nonselective proteins. A green diamond indicates Ald6, a selective cargo of autophagosomes. R^2^ indicates correlation coefficients for regression lines. (F) Enlargement of the square in (E). (1) Pyk1, (2) Yef3, (3) Hsc82, (4) Eft1, (5) Ald6, (6) Ape1, (7) Fas1, (8) Tif1, (9) Pfk1, (10) Fas2, (11) Gdh1, (12) Met6, (13) Tef4, (14) Pfk2, and (15) Asc1. (G) Quantification of the ratios of Pfk and Adh proteins in the cytoplasmic fraction (S15) and in a vacuole fraction containing autophagic bodies (P15). Vacuolar proteinase–deficient mutants were used to prevent degradation of autophagic bodies. Asterisk indicates *P*<0.05 (N = 3, two-tailed Student’s *t*-test).

Data sets were generated for AP fractions from *ypt7*Δ, *ypt7*Δ*atg1*Δ, and *ypt7*Δ*atg11*Δ cells, and for cytosol (100,000×*g* supernatant) prepared from *ypt7*Δ cells, in three independent experiments (Table S1). We subjected these data sets to PCA as described in Materials and Methods (Tables S2–S4). The scores of the first two PCs were plotted on a scatter diagram (Fig. 4A). The first principal component (PC1) corresponded to spectral counts, and the second principal component (PC2) reflected localization of proteins: cytoplasmic proteins had positive PC2 values (Fig. 4A, blue dots), whereas mitochondrial and plasma-membrane proteins had negative values (Fig. 4A, red and black dots). Because mitochondrial and plasma-membrane proteins were detected in the *ypt7*Δ*atg1*Δ AP fraction (Table S8), proteins with negative values were considered to represent contamination. This PCA enables us to discriminate autophagosome cargo from contaminating mitochondrial and plasma-membrane proteins.

Next, we plotted the spectral counts of mitochondrial proteins from the AP fractions of *ypt7*Δ and *ypt7*Δ*atg1*Δ cells. This diagram indicates that no mitochondrial proteins were concentrated in the *ypt7*Δ AP fraction (Fig. 4B). By contrast, the scatter plot of cytoplasmic proteins revealed that several proteins were concentrated in the *ypt7*Δ AP fraction (Fig. 4C); these included Pyk1 (pyruvate kinase), Yef3 (subunit of a translational elongation factor), Hsc82 (cytoplasmic chaperone of the Hsp90 family), Eft1 (elongation factor 2), Ald6, and prApe1. Subsequently, we compared the spectral counts of cytoplasmic proteins from the *ypt7*Δ and *ypt7*Δ*atg11*Δ AP fractions (Fig. 4D), revealing that only prApe1 is concentrated in the *ypt7*Δ AP fraction. This result reflects the fact that prApe1 is enclosed in autophagosomes by a mechanism dependent on Atg11 [19], but Ald6 is enclosed in autophagosomes in an Atg11-independent manner [4]. This result also implies that Pyk1, Yef3, Hsc82, and Eft1 are concentrated in autophagosomes by a mechanism independent of Atg11.

Next, we performed PCA on cytoplasmic proteins of AP fractions from *ypt7*Δ *ypt7*Δ*atg1*Δ, and *ypt7*Δ*atg11*Δ cells (Table S5 and Fig. S2). Because that Tdh3 and prApe1 had large impacts on the results of PCA, we omitted the Tdh3 and prApe1 data from subsequent data-mining analysis. The first and second PCs were calculated using the modified data sets (Table S6) and plotted (Fig. 4E–F). We found that nonselective cargo proteins (Adh1, Pdc1, Fba1, Tpi1, and Pho8Δ60) [4], [20] were distributed along a regression line with a positive slope (black diamonds in Fig. 4E–F). By contrast, Ald6 and ribosomal proteins yielded negative values and were distributed along another regression line with a negative slope (green and red diamonds in Fig. 4E–F). In addition to the ribosomal proteins, ribosome-associated proteins, such as Eft1, Tif1 (translation initiation factor), Tef4 (subunit of translational elongation factor), and Asc1 (component of 40S ribosomal subunit) were distributed along the same regression line (Fig. 4E), suggesting that these proteins are enclosed in autophagosomes along with ribosomes.

In addition to the ribosomal and ribosome-associated proteins, several cytoplasmic proteins were plotted along the regression line with negative slope; for example, Hsc82, Fas1/2 (subunits of fatty-acid synthetase), Pfk1/2 (subunits of phosphofructokinase), Gdh1 (NADP(+)-dependent glutamate dehydrogenase), and Met6 (methionine biosynthesis) were concentrated in the *ypt7*Δ AP fraction (Fig. 4E–F), suggesting that these proteins are also selective cargoes. It is noteworthy that Fas1 and Fas2, components of fatty acid synthetase, were identified here: because Fas1 has been used to isolate *aut* (autophagocytosis) mutants [21], the fatty acid–synthase complex is a strong candidate for a selective cargo of autophagosomes.

### Validation of a Candidate for Selective Cargoes

We next examined whether the Pfk1/2 complex, a key enzyme of glycolysis, was selectively transported to the vacuole by autophagy. Autophagic bodies, which are produced by fusion of autophagosomes with the vacuole, accumulate in *pep4*Δ cells upon starvation [22]. Therefore, the cargo of autophagic bodies is equivalent to that of autophagosomes. We fractionated lysates from starved *pep4*Δ and *pep4*Δ*atg7*Δ cells into S15 (cytoplasm) and P15 (vacuole) fractions, and subjected the resulting fractions to immunoblot analysis (Fig. S3). In this experiment, we used Adh (alcohol dehydrogenase) as a representative nonselective cargo protein [4]. Quantification of Pfk and Adh signals in each fraction revealed that the Pfk/Adh ratio of the vacuole fraction, which contained autophagic bodies, was significantly higher than that of the cytoplasmic fraction (Fig. 4G). Thus, the Pfk1/2 complex is enriched in autophagosomes and then transported to the vacuole.

## Discussion

In this study, we first developed a simple and easy method to monitor intact autophagosomes *ex vivo* by detecting GFP-prApe1 dots inside autophagosomes. This method facilitated optimization of a biochemical procedure for fractionating autophagosomes. LC-MS/MS and subsequent statistical analyses identified autophagosome cargo proteins, including candidates for novel selective cargoes in addition to known selective cargoes. Among these candidates, we showed that the Pfk1/2 complex is a selective cargo of autophagy.

Besides Pfk1/2, Fas1/2 are candidates of cytoplasmic proteins selectively enclosed in autophagosomes (Fig. 4E–F). Degradation of Fas1 has been used as a marker in isolation of autophagy mutants [21]. Although fatty-acid synthase is degraded in the vacuole during starvation, a significant decrease in the level of fatty-acid synthase is not observed under these conditions [23]. This result was reproducible in our hands (data not shown). Thumm *et al*. postulated that this might be because synthesis of fatty-acid synthase is accompanied by its degradation [21].

In animal cells, p62 and NBR1, both of which are selective cargo proteins [24], might be used as markers for visualization of autophagosomes. The system described here would be broadly applicable to analysis of autophagosome cargo in other eukaryotes.

We found a number of candidates for selective cargoes enclosed in autophagosomes by an Atg11-independent mechanism (Fig. 4E–F). The next step of our study is to validate candidate proteins and to elucidate the mechanism conferring the selectivity. The methodology described here will pave the way to analyze the mechanism.

## Supporting Information

Figure S1 **Autophagosomes in** ***ypt7*** **Δ cells.** Localization of GFP-prApe1. *ypt7*Δ cells were incubated in nitrogen starvation medium for 4.5 hours. Arrowheads indicate weaker GFP-prApe1 dots, which correspond to autophagosomes. Bar represents 5 µm.
(TIF)

Figure S2 **Principal component analysis of AP fractions from** ***ypt7*** **Δ,** ***ypt7*** **Δ** ***atg1*** **Δ, and** ***ypt7*** **Δ** ***atg11*** **Δ cells.** The first and second principal components (PC1 and PC2) are plotted. Green diamonds indicate prApe1 and Ald6, both of which are previously reported selective cargoes of autophagosomes. Black diamonds indicate nonselective cargoes (Onodera and Ohsumi, 2004). Blue diamonds indicate other cytoplasmic proteins.
(TIF)

Figure S3 **Immunoblot analysis of cytoplasmic and vacuole fractions.** Cells grown in YEPD medium were starved in SD(-N) medium for 4.5 hours. Cells were converted to spheroplasts, and then physically disrupted with 3.0 µm-pore polycarbonate filters. After debris was removed, cell lysates were centrifuged at 15,000 x *g* for 15 minutes at 4°C and separated into supernatant (S15) and pellet (P15) fractions. The P15 fractions were washed once with lysis buffer. As a representative of cytoplasm, the S15 fraction of *pep4*Δ*atg7*Δ cells was serially diluted (lanes 1–3). To detect Adh, one-eighth volume of the S15 fraction was loaded for each lane (lanes 1–3). Serially diluted P15 fractions were loaded as follows: *pep4*Δ cells: lanes 4, 6, and 8; *pep4*Δ*atg7*Δ cells: lanes 5, 7, and 9. Intensities of chemiluminescent signals were quantified using Image Gauge software (Fujifilm, Japan). To calculate Pfk/Adh ratios in cytoplasm (lanes 1–3), signals for Pfk1/2 and Adh were quantified by subtracting the corresponding background intensities from the band intensities. To calculate the ratios in autophagic bodies, Pfk and Adh signals of the *pep4*Δ P15 fractions were adjusted by subtracting the corresponding signals of *pep4*Δ*atg7*Δ P15 fractions. Calculated ratios were normalized to the ratio of lane 1.
(TIF)

Table S1 **Complete data obtained from mass spectrometric analysis (673 proteins).**
(XLSX)

Table S2 **Data used for principal component analyses (378 proteins;** ***ypt7*** **Δ_AP in Table S1>0).**
(XLSX)

Table S3 **Principal component analysis for** **Figure 4A** **(378 proteins).**
(XLSX)

Table S4 **Protein localization manually annotated (for** **Figure 4A** **).**
(XLSX)

Table S5 **Principal component analysis for Figure S2 (186 proteins; PC2 scores in Table S4> = 0).**
(XLSX)

Table S6 **Principal component analysis for** **Figure 4E and 4F** **(184 proteins; Tdh3 and Ape1 are** **omitted).**
(XLSX)

Table S7 **Top 40 proteins identified in the AP fraction of** ***ypt7*** **Δ cells.**
(XLSX)

Table S8 **Top 40 proteins identified in the AP fraction of** ***ypt7*** **Δ** ***atg1*** **Δ cells.**
(XLSX)

Appendix S1 **Supplemental Materials and Methods for LC-MS/MS spectrometry.**
(DOC)
