# Supplementary figures and images for: Proteomic Profiling of Autophagosome Cargo in Saccharomyces cerevisiae
Source: PLoS One. 2014 Mar 13;9(3):e91651. doi: 10.1371/journal.pone.0091651 (PMC3953483; doi:10.1371/journal.pone.0091651)

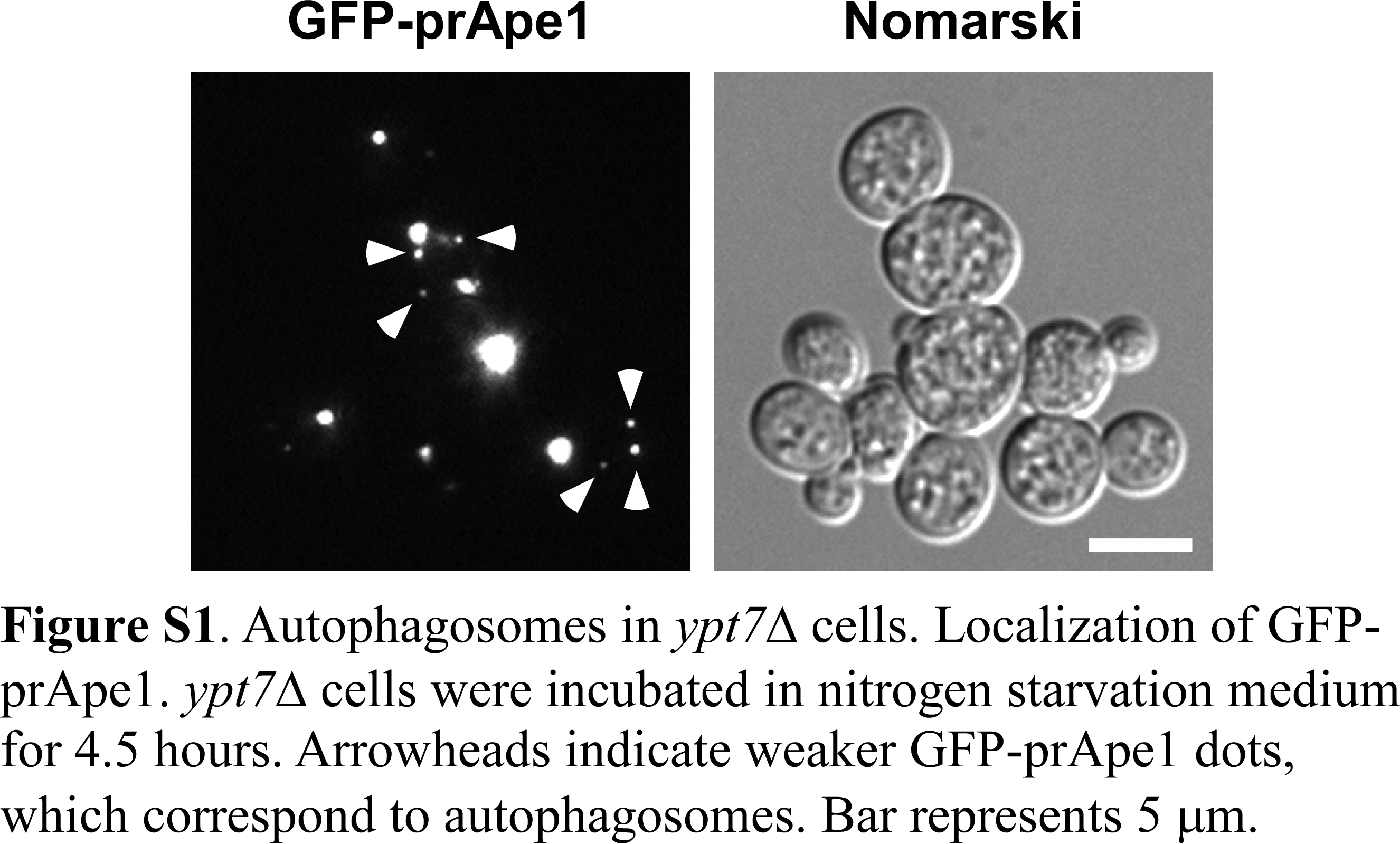

Supplement: Figure S1 — Autophagosomes in ypt7 Δ cells. Localization of GFP-prApe1. ypt7Δ cells were incubated in nitrogen starvation medium for 4.5 hours. Arrowheads indicate weaker GFP-prApe1 dots, which correspond to autophagosomes. Bar represents 5 µm. (TIF) [file pone.0091651.s001.tif]

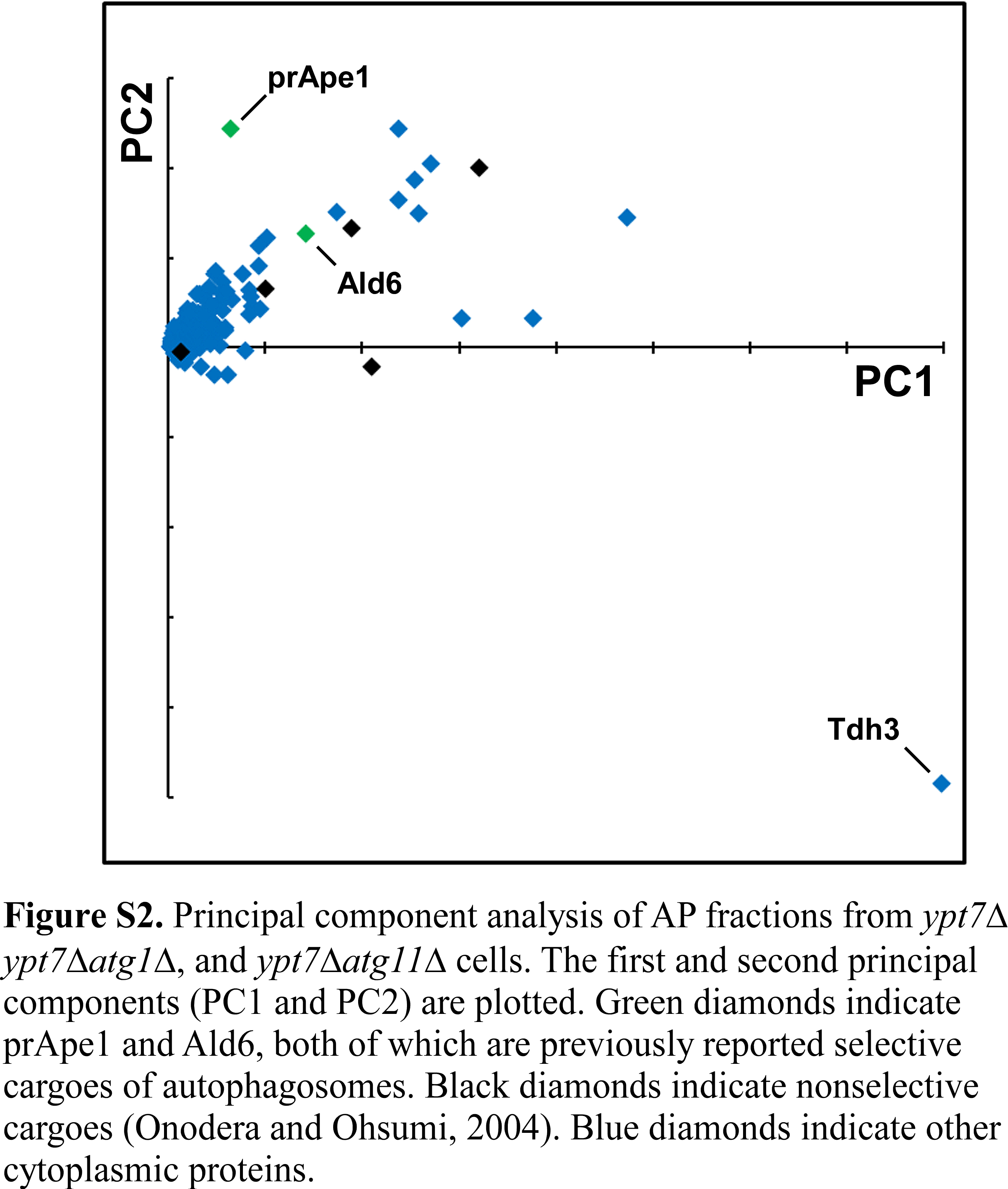

Supplement: Figure S2 — Principal component analysis of AP fractions from ypt7 Δ, ypt7 Δ atg1 Δ, and ypt7 Δ atg11 Δ cells. The first and second principal components (PC1 and PC2) are plotted. Green diamonds indicate prApe1 and Ald6, both of which are previously reported selective cargoes of autophagosomes. Black diamonds indicate nonselective cargoes (Onodera and Ohsumi, 2004). Blue diamonds indicate other cytoplasmic proteins. (TIF) [file pone.0091651.s002.tif]

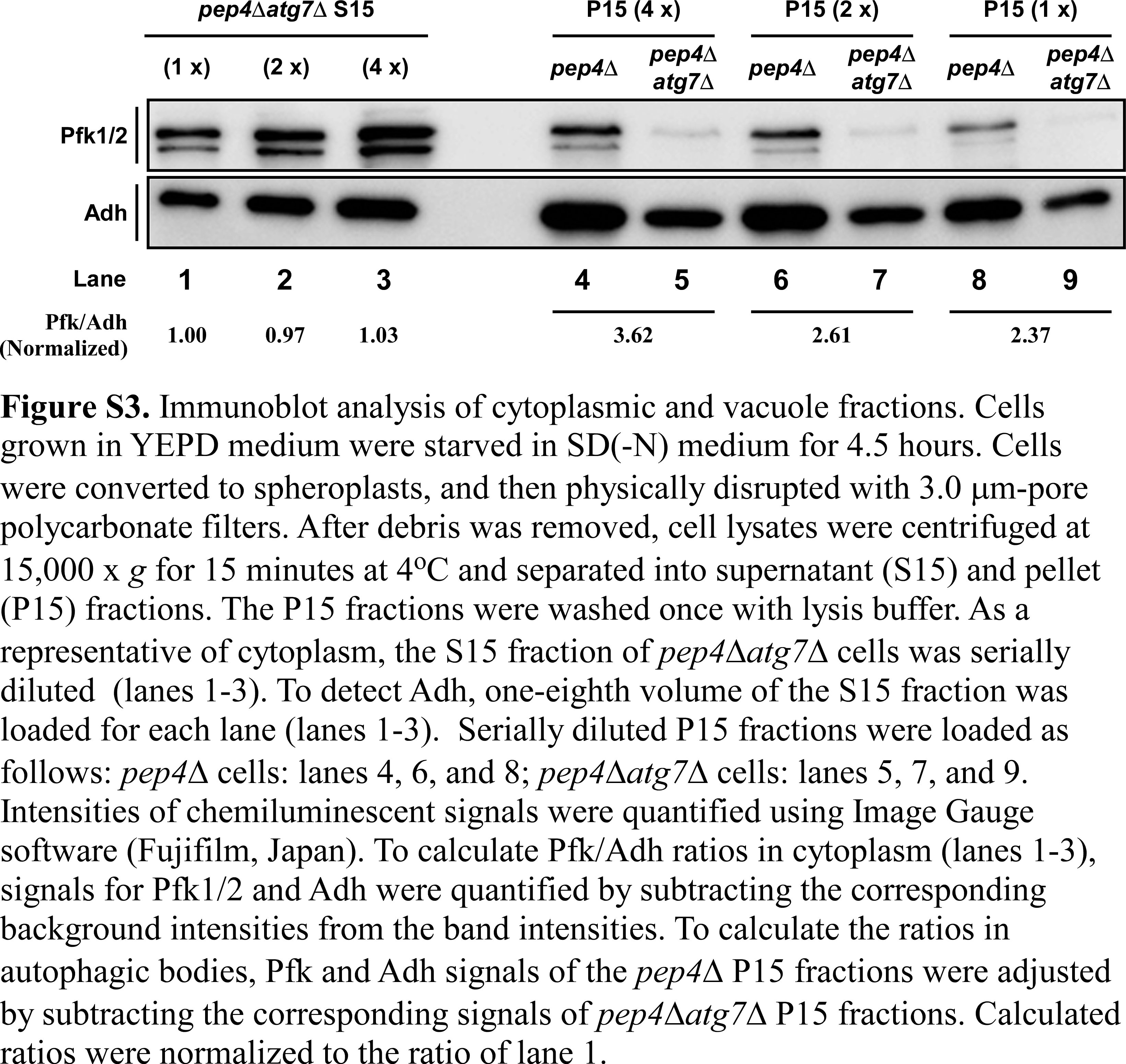

Supplement: Figure S3 — Immunoblot analysis of cytoplasmic and vacuole fractions. Cells grown in YEPD medium were starved in SD(-N) medium for 4.5 hours. Cells were converted to spheroplasts, and then physically disrupted with 3.0 µm-pore polycarbonate filters. After debris was removed, cell lysates were centrifuged at 15,000 x g for 15 minutes at 4°C and separated into supernatant (S15) and pellet (P15) fractions. The P15 fractions were washed once with lysis buffer. As a representative of cytoplasm, the S15 fraction of pep4Δatg7Δ cells was serially diluted (lanes 1–3). To detect Adh, one-eighth volume of the S15 fraction was loaded for each lane (lanes 1–3). Serially diluted P15 fractions were loaded as follows: pep4Δ cells: lanes 4, 6, and 8; pep4Δatg7Δ cells: lanes 5, 7, and 9. Intensities of chemiluminescent signals were quantified using Image Gauge software (Fujifilm, Japan). To calculate Pfk/Adh ratios in cytoplasm (lanes 1–3), signals for Pfk1/2 and Adh were quantified by subtracting the corresponding background intensities from the band intensities. To calculate the ratios in autophagic bodies, Pfk and Adh signals of the pep4Δ P15 fractions were adjusted by subtracting the corresponding signals of pep4Δatg7Δ P15 fractions. Calculated ratios were normalized to the ratio of lane 1. (TIF) [file pone.0091651.s003.tif]
