# Supplementary material for: Proteomic Profiling of Autophagosome Cargo in Saccharomyces cerevisiae
Source: PLoS One. 2014 Mar 13;9(3):e91651. doi: 10.1371/journal.pone.0091651 (PMC3953483; doi:10.1371/journal.pone.0091651)
Supplement: Appendix S1 — Supplemental Materials and Methods for LC-MS/MS spectrometry. (DOC) [file pone.0091651.s012.doc]

**Appendix S1. Supplemental Materials and Methods**

**LC-MS/MS spectrometry**

*Tryptic digestion*

Protein digestion was performed essentially according to the tube-gel digestion protocol described previously [1]. In brief, 5 μg of protein sample in 13 μL of 100 mM ammonium bicarbonate (ABC) solution, 6 μL of 30% acrylamide solution, 0.7 μL of 10% ammonium persulfate, and 0.3 μL of TEMED were mixed in a 0.5-mL Eppendorf tube, and the polymerization reaction was carried out for 30 min at room temperature. The obtaining gel strip was cut into small pieces (less than 1 mm^3^) and washed twice with 100 mM ABC solution containing 45% acetonitrile for 10 min, with vortexing in a micro-tube mixer. The gel pieces were then subjected to the standard in-gel digestion in a methylpentene polymer tube. All of the subsequent reactions were carried out in an Eppendorf Thermomixer Comfort at 850 rpm. Proteins were reduced with 40 μL of 10 mM TCEP at 37 °C for 45 min, and then alkylated with 40 μL of 50 mM iodoacetamide at room temperature in the dark for 60 min. After three washes with 100 mM ABC solution and dehydration with acetonitrile, proteolytic digestion was performed with 50 μL of 0.01 μg/μL trypsin dissolved in 100 mM ABC solution; digestion reactions were incubated at 37°C overnight. Extracted peptides were mixed with 40 μL of 1% TFA solution and subjected to LC-MS/MS analysis.

*LC-MS/MS*

We adopted label-free semi-quantitation using spectral counting by LC-MS/MS to perform a global proteomic analysis. The digested samples were analyzed in triplicate by LC-MS/MS using reversed-phase liquid chromatography (RP-LC) interfaced with a LTQ-Orbitrap hybrid mass spectrometer (Thermo Fisher Scientific) via a nanoelectrospray device, as described in detail previously [2]. Briefly, the RP-LC system consisted of a peptide Cap-Trap cartridge (2.0 × 0.5 mm) and a capillary separation column (L-column Micro, 50 × 0.2 mm L-C18, 3 μm, 12 nm) connected an emitter tip (FortisTip) to the outlet. An autosampler (HTC-PAL, CTC Analytics) loaded aliquots of samples onto the trap, which was then washed with solvent A (2% acetonitrile [aq.] containing 0.1% formic acid) to concentrate peptides on the trap and for desalting. Subsequently, the trap was connected in series to the separation column, and the whole column was developed for 70 min in linear-gradient mode from 5–40% solvent B (90% acetonitrile [aq.] containing 0.1% formic acid) at a flow rate of 1 μL/min. The mass spectrometer was operated in data-dependent mode in order to automatically switch between one high-resolution MS scan (resolution, 30,000; scan range, m/z 400–1600) by the Orbitrap and up to four concurrent MS/MS scans in the LTQ for the four most intense peaks selected from each MS scan (ion selection threshold, 1000 counts). Automatic gain control was set to 500,000 for the Orbitrap MS scans and 10,000 for the LTQ MS/MS scans. MS scans were acquired in profile mode and MS/MS scans were acquired in centroid mode. General MS conditions were as follows: ESI voltage, 3.0 kV; no sheath or auxiliary gas flow; ion transfer tube temperature, 200°C; collision energy, 35%. An activation q-value of 0.25 and an activation time of 30 ms were applied for MS/MS acquisitions. The sets of acquired high-resolution MS and MS/MS spectra for peptides were converted to individual DTA files using the Bioworks software, and these DTA files were merged into Mascot generic format files for database searching.

*Identification of proteins*

The Mascot software (version 2.1.1, Matrix Science) was used in database searches against 6,903 entries for *Saccharomyces cerevisiae* in the UniProtKB/Swiss-Prot database (Release 57.10). Peptide mass tolerance was 20 ppm, fragment mass tolerance was 0.8 Da, and up to two missed cleavages were allowed for errors in trypsin specificity. Carbamidomethylation of cysteines was taken as a fixed modification, and methionine oxidation as a variable modification. The false-positive rates (FDRs) for protein identification were estimated using a decoy database created by reversing the protein sequences in the original database; the estimated FDR of peptide matches was 0.47% under protein-score threshold conditions (*P* < 0.005).

*Spectral counting method*

For semi-quantitative analysis, protein identification from individual Mascot search results was integrated using the Scaffold software (version 2.02.03, Proteome Software). The number of high-confidence peptide spectra (Mascot ion score*,* *P* < 0.005) was used as the spectral-count value. All proteins with more than two peptide spectra in a single LC-MS/MS analysis were considered for protein quantification using spectral counting. The averaged values based on the triplicate analyses were estimated as the spectral-count values of each protein in individual samples.

**Supplemental references**

1. Lu X, Zhu H (2005) Tube-gel digestion: a novel proteomic approach for high throughput analysis of membrane proteins. Mol Cell Proteomics 4: 1948-1958.

2. Kawase H, Fujii K, Miyamoto M, Kubota KC, Hirano S, et al. (2009) Differential LC-MS-based proteomics of surgical human cholangiocarcinoma tissues. J Proteome Res 8: 4092-4103.
